# Automated coronary calcium scoring using deep learning with multicenter external validation

**DOI:** 10.1038/s41746-021-00460-1

**Published:** 2021-06-01

**Authors:** David Eng, Christopher Chute, Nishith Khandwala, Pranav Rajpurkar, Jin Long, Sam Shleifer, Mohamed H. Khalaf, Alexander T. Sandhu, Fatima Rodriguez, David J. Maron, Saeed Seyyedi, Daniele Marin, Ilana Golub, Matthew Budoff, Felipe Kitamura, Marcelo Straus Takahashi, Ross W. Filice, Rajesh Shah, John Mongan, Kimberly Kallianos, Curtis P. Langlotz, Matthew P. Lungren, Andrew Y. Ng, Bhavik N. Patel

**Affiliations:** 1 Department of Computer Science, Stanford University School of Medicine, Stanford, CA USA; 2 Bunkerhill, Palo Alto, CA USA; 3 Department of Pediatrics, Stanford University School of Medicine, Stanford, CA USA; 4 Department of Radiology, Stanford University School of Medicine, Stanford, CA USA; 5 Division of Cardiovascular Medicine and Stanford Prevention Research Center, Department of Medicine, Stanford University School of Medicine, Palo Alto, CA USA; 6 Department of Radiology, Duke University Medical Center, Durham, NC USA; 7 Lundquist Institute at Harbor-UCLA Medical Center, Torrance, CA USA; 8 Diagnósticos da América SA (Dasa), Alphaville Barueri, SP Brazil; 9 Department of Diagnostic Imaging, Universidade Federal de São Paulo (Unifesp), São Paulo, SP Brazil; 10 Department of Radiology, MedStar Georgetown University Hospital, Washington, DC USA; 11 Radiology Service, VA Palo Alto Health Care System, Palo Alto, CA USA; 12 Department of Radiology and Biomedical Imaging and Center for Intelligent Imaging, University of California, San Francisco, School of Medicine, San Francisco, CA USA; 13 Department of Radiology, Mayo Clinic, Scottsdale, AZ USA

**Keywords:** Medical imaging, Computer science

## Abstract

Coronary artery disease (CAD), the most common manifestation of cardiovascular disease, remains the most common cause of mortality in the United States. Risk assessment is key for primary prevention of coronary events and coronary artery calcium (CAC) scoring using computed tomography (CT) is one such non-invasive tool. Despite the proven clinical value of CAC, the current clinical practice implementation for CAC has limitations such as the lack of insurance coverage for the test, need for capital-intensive CT machines, specialized imaging protocols, and accredited 3D imaging labs for analysis (including personnel and software). Perhaps the greatest gap is the millions of patients who undergo routine chest CT exams and demonstrate coronary artery calcification, but their presence is not often reported or quantitation is not feasible. We present two deep learning models that automate CAC scoring demonstrating advantages in automated scoring for both dedicated gated coronary CT exams and routine non-gated chest CTs performed for other reasons to allow opportunistic screening. First, we trained a gated coronary CT model for CAC scoring that showed near perfect agreement (mean difference in scores = −2.86; Cohen’s Kappa = 0.89, *P* < 0.0001) with current conventional manual scoring on a retrospective dataset of 79 patients and was found to perform the task faster (average time for automated CAC scoring using a graphics processing unit (GPU) was 3.5 ± 2.1 s vs. 261 s for manual scoring) in a prospective trial of 55 patients with little difference in scores compared to three technologists (mean difference in scores = 3.24, 5.12, and 5.48, respectively). Then using CAC scores from paired gated coronary CT as a reference standard, we trained a deep learning model on our internal data and a cohort from the Multi-Ethnic Study of Atherosclerosis (MESA) study (total training *n* = 341, Stanford test *n* = 42, MESA test *n* = 46) to perform CAC scoring on routine non-gated chest CT exams with validation on external datasets (total *n* = 303) obtained from four geographically disparate health systems. On identifying patients with any CAC (i.e., CAC ≥ 1), sensitivity and PPV was high across all datasets (ranges: 80–100% and 87–100%, respectively). For CAC ≥ 100 on routine non-gated chest CTs, which is the latest recommended threshold to initiate statin therapy, our model showed sensitivities of 71–94% and positive predictive values in the range of 88–100% across all the sites. Adoption of this model could allow more patients to be screened with CAC scoring, potentially allowing opportunistic early preventive interventions.

## Introduction

Cardiovascular disease (CVD) is the leading cause of death globally, responsible for approximately 17.9 million deaths in 2016^1^. Heart disease, the most common manifestation of CVD, remains the most common cause of mortality in the United States, accounting for over 655,000 deaths in 2016^2^. Coronary artery disease (CAD), the most common type of heart disease, was responsible for 365,914 deaths in the United States in 2017^3^. Coronary events are estimated to occur every 25 s with a death from the event occurring every minute in the United States^4^. Risk assessment is the cornerstone for primary prevention of CVD and coronary events, particularly as the long asymptomatic latency period of CAD provides a window of opportunity for early preventive intervention^5^. Moreover, treatment decisions and guidelines, such as initiation of statins and anti-hypertensives are based on 10-year risk estimations using risk scores^6–9^. The American College of Cardiology and the American Heart Association currently recommend use of the Pooled Cohort Equations to guide risk assessment and tailor preventive therapies. However, these and other risk prediction tools remain imperfect and have significant limitations including poor performance across diverse populations^10^. Furthermore, many patients fall into an indeterminant or intermediate risk categories, requiring use of additional noninvasive assessment for proper risk stratification^7,11,12^.

Coronary artery calcium (CAC) scoring using computed tomography (CT) is one of the most powerful independent noninvasive predictors of CAD and has been shown to discriminate well across diverse populations^11,13–15^. Coronary calcium burden on cardiac CTs, expressed as Agatston scores, has been shown to be more prognostic and clinically useful when treatment decisions are unclear for patients categorized as intermediate risk using traditional risk models^5,8,11^. Despite the clinical value of CAC scoring, the current clinical practice for CAC assessment arguably has two major limitations. First, CAC scoring using gated coronary CT scans, the gold standard, often require significant resources, which may not be operationally feasible at small centers. These include capital intensive CT machines and specialized monitoring (e.g. electrocardiogram (ECG) gating and potential administration of beta-blockers)^16,17^. After image acquisition, specialized software on independent workstations, accredited 3D imaging labs, and specialized technologists to separately perform the task of coronary artery segmentations and calcium burden quantification is typically required. This clinical workflow paradigm often results in delay of reporting the official CAC score. Moreover, some imaging centers may not be able to offer coronary calcium risk assessment due to the lack of aforementioned human and capital resources. A second, and perhaps a far more significant, limitation is the millions of patients who undergo routine, non-gated chest CTs for non-cardiac indications (e.g., lung cancer screening, infection, etc.) which demonstrate coronary artery disease but whose presence is not routinely reported nor quantified thereby missing potential opportunities for early disease prevention^18^. Automation of CAC scoring has the potential to address these shortcomings in current clinical practice.

Recent advances in deep learning techniques in image recognition and image segmentation have motivated research in applying deep learning applications to automated analysis of medical imaging^19–22^. Though many semi-automated and few automated calcium scoring methods using gated CTs have been proposed in the literature, to our knowledge, no study to date has reported fully automated vessel-specific coronary calcium scoring using an end-to-end deep learning architecture with prospective validation and multi-center external validation^23–25^. Additionally, while some studies^26–32^ have reported the feasibility of automated methods to quantify coronary calcium from non-gated unenhanced chest CTs, they are either not end-to end^27,28^ or do not use deep learning methods^27,30^. Most importantly, for validation, none of these studies^26–32^ curated a dataset comprised of a strong clinical reference standard for ground truth, which calls into question the clinical claims and require further validation.

In this work, we hypothesized that deep learning models can reliably provide accurate and rapid coronary artery calcium scoring using both gated coronary calcium and routine non-gated chest CTs. Thus, the purpose of our study was to develop two deep learning models that automatically quantify vessel-specific CAC score using gated coronary calcium and non-gated chest CTs. For the non-gated model training and testing, we used a strong reference standard using calcium scores derived from gated studies. We externally validated our non-gated model on paired gated and non-gated datasets from four major geographically disparate health systems.

## Results

In this study, two deep learning models were developed that automate CAC scoring using gated unenhanced coronary CT and non-gated unenhanced chest CT, respectively. Please refer to the  methods section for complete details regarding the datasets used, architecture details, deep learning model training, and reference standard. Tables 1 and 2 show the cohort demographic information and statistics from our internal and external sites.

**Table 1:** Cohort demographics and statistics for gated model.

Characteristic	Retrospective gated coronary CT cohort				Prospective gated coronary CT cohort
	Training	Validation	Test	Total	Test
No. of individuals (%F)	697 (51)	78 (56)	79 (43)	854 (51)	55
No. of exams	708	79	79	866	55
Mean age (range [±standard deviation])	56.9 (22–86[±12.5])	57.2 (18–82[±12.5])	56.5 (27–85[±11.8])	56.9 (18–86[±12.4])	56.7 (35–75[±10.5])
% Flash scanner (vs. Force)	50.4	44.3	57.0	50.5	45.5
% GE Scanner (vs. Siemens)	N/A	N/A	N/A	N/A	N/A
CAC Score Bucket					
I	346	40	40	426	25
II	55	9	3	67	10
III	115	14	16	145	11
IV	91	9	8	108	5
V	99	8	13	120	4

**Table 2:** Cohort demographics and statistics for non-gated model.

Characteristic	Retrospective non-gated coronary CT Cohort								External non-gated validation cohorts			
	Stanford				MESA							
	Training	Validation	Test	Total	Training	Validation	Test	Total	Site 1	Site 2	Site 3	Site 4
Patient sex												
M	87	11	24	122	67	11	21	99	22	53	46	61
F	64	11	18	93	59	11	24	94	0	22	25	74
N/A^a^	0	0	0	0	37	1	1	39	0	0	0	0
No. of exams	151	22	42	215	163	23	46	232	22	75	71	135
Mean age (range [± standard deviation])	60.9 (18–80[±9.8])	60.6 (31–82[±12.1])	60.6 (47–73[±7.5])	60.8 (18–82[±9.6])	70.0 (54–111[±9.0])	65.0 (55–80[±7.3])	65.9 (55–91[±9.0])	67.8 (54–111[±9.0])	64.6 (38–76[±10.1]	62.4 (31–88[±13.3])	66.5 (43–83[±9.0])	N/A
Median time interval^b^	8.0	8.2	3.3	6.2	0.0	0.0	0.0	0.0	2.7	1.0	5.6	2.4
Scanner												
GE	55	9	20	84	40	40	0	0	22	39	0	131
Siemens	90	13	20	123	123	123	23	46	0	11	71	3
Other	6	0	2	8	0	0	0	0	0	25	0	1
CAC Score Bucket												
I	59	12	18	89	48	6	7	61	4	22	28	52
II	12	5	3	20	12	3	4	19	1	4	4	11
III	35	3	5	43	38	3	14	55	3	17	13	22
IV	24	1	10	35	34	7	13	54	4	16	14	32
V	21	1	6	28	30	4	8	42	11	16	12	18

^a^Information was not available due to site specific patient privacy rules.

^b^Median time interval (months) between non-gated routine chest CT and gated coronary CT for each test set.

### Retrospective gated coronary model

**Fig. 1:**
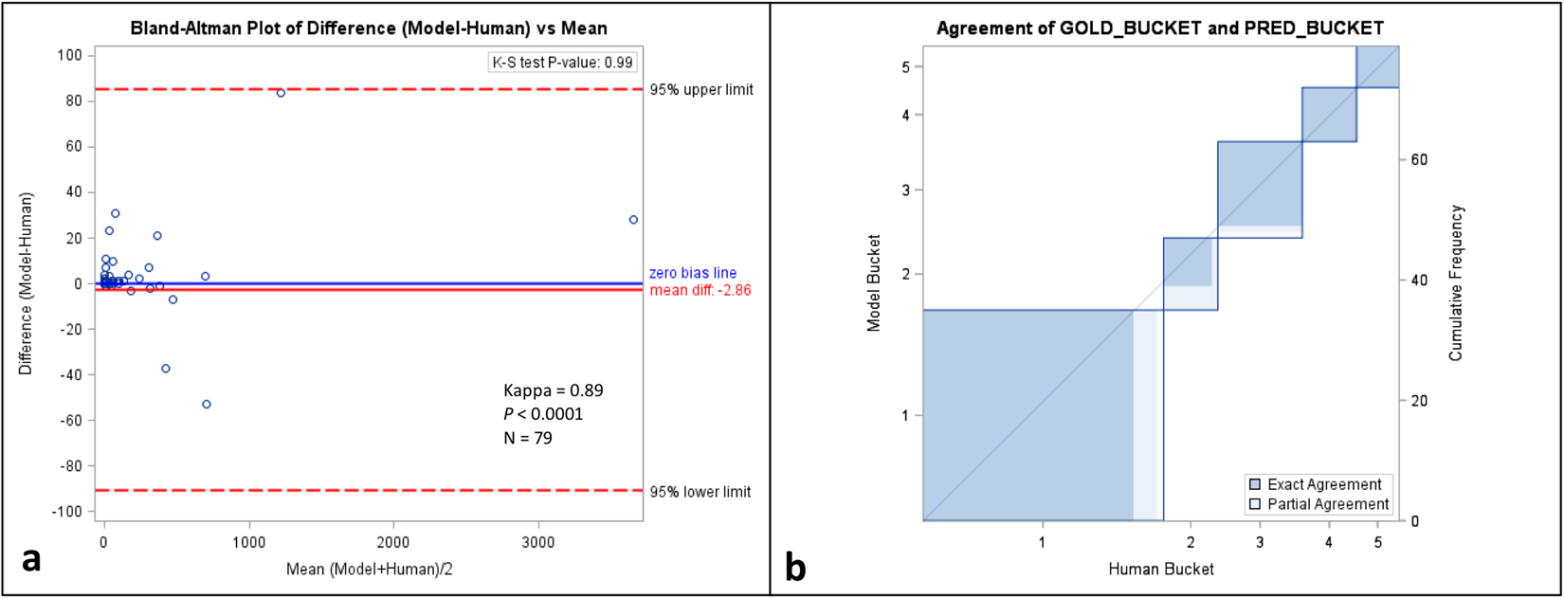
Comparison of automated scoring using deep learning and manual scoring in a retrospective cohort. Bland–Altman plot (**a**) and Cohen’s Kappa statistic agreement plot (**b**) comparing retrospective automated scoring of gated AI model to manual scoring of CAC using gated coronary CT exams. Please refer to Supplementary Fig. 1 for a zoomed version of the Bland–Altman plot.

**Fig. 2:**
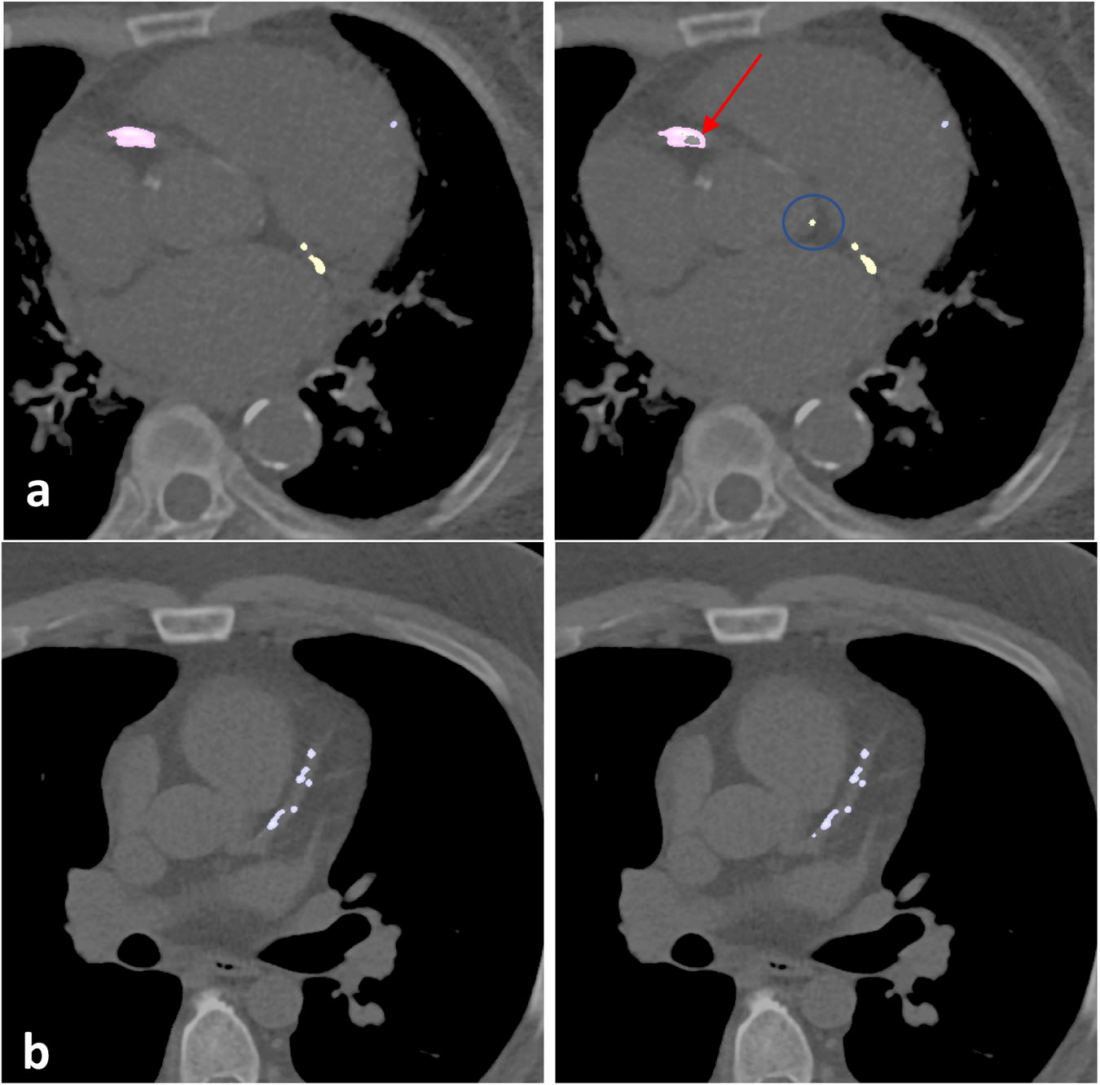
Qualitative comparison between automated and manual CAC scoring. Coronary artery segmentation (manual on left; automated on right) in two different patients (**a**, **b**) shows qualitatively similar performance between automated and manual methods of CAC scoring. Note the false positive by model identifying coronary cusp calcification (blue circle). Remainder of aortic root calcification is accurately ignored by the model. Red arrow shows an area of false negative within an area of right coronary artery calcification.

Using a test set of 79 studies in 79 patients (Table 1), Bland–Altman analysis showed little difference in individual vessel-specific scores between manually derived scores and those predicted by the model (mean difference [95% confidence interval (CI)]: −2.86 [−88.49, 82.71]) (Figs. 1 and 2 and Supplementary Fig. 1). Biases [95% CI] for each vessel were −3.10 [−33.80, 27.59], 4.57 [−23.94, 33.08], −1.99 [−27.06, 23.09], and −2.37 [−75.51, 70.77] for left main (LCA), left anterior (LAD), left circumflex (LCX), and right coronary (RCA) arteries, respectively. Qualitatively, color coded masks of coronary calcium generated by the model and those manually appeared similar with small differences (Fig. 2). Kolmogorov–Smirnov (K–S) test showed no statistically significant difference in the distribution of CAC scores using the two methods (*P* = 0.99). When bucket scores (I–V, for Agatston scores of 0, 1–10, 11–100, 101–400, >400, respectively) were compared using Cohen’s Kappa statistic, there was almost perfect agreement between the two methods (Kappa = 0.89, *P* < 0.0001).

### Prospective validation of gated coronary model

**Fig. 3:**
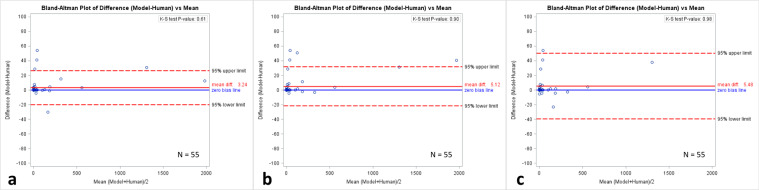
Comparison of automated scoring using deep learning and manual scoring in a prospective cohort. Bland–Altman plots comparing prospective automated scoring of gated AI model to manual scoring of CAC by three different (**a**–**c**) technologists using gated coronary CT exams. Please refer to Supplementary Fig. 2 for a zoomed version of the Bland–Altman plot.

**Fig. 4:**
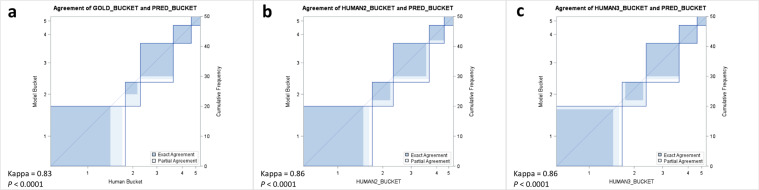
Comparison of automated and manual scoring in a prospective cohort. Cohen’s Kappa statistic agreement plots comparing automated scoring to manual scoring by three different (**a**–**c**) technologists using gated coronary CT exams.

A prospective trial of 55 patients (Table 1) who were referred to our department for CAC scoring was performed to compare automated scores to those derived manually by three technologists. Bland–Altman analysis showed little difference when comparing automatic to the three manually derived individual vessel-specific scores (mean difference in model from humans: 3.24, 5.12, and 5.48, respectively) (Figs. 3, 4, and Supplementary Fig. 2). K–S test showed no statistically significant difference in the distribution of automated and manually derived CAC scores (*P*-values = 0.61, 0.90, and 0.98, respectively). Zero-inflated Poisson regression test showed no statistically significant difference in vessel-specific CAC scores (*P* = 0.82). When bucket scores (I–V) were compared using Cohen’s Kappa statistic, there was almost perfect agreement between scores derived using the deep learning model and those obtained manually (Kappa = 0.83, 0.86, and 0.86, respectively, *P* < 0.0001). Average time for automated CAC scoring using a graphics processing unit (GPU) was 3.5 ± 2.1 s compared to 261 s for manual scoring.

### Non-gated chest CT model: internal validation

**Fig. 5:**
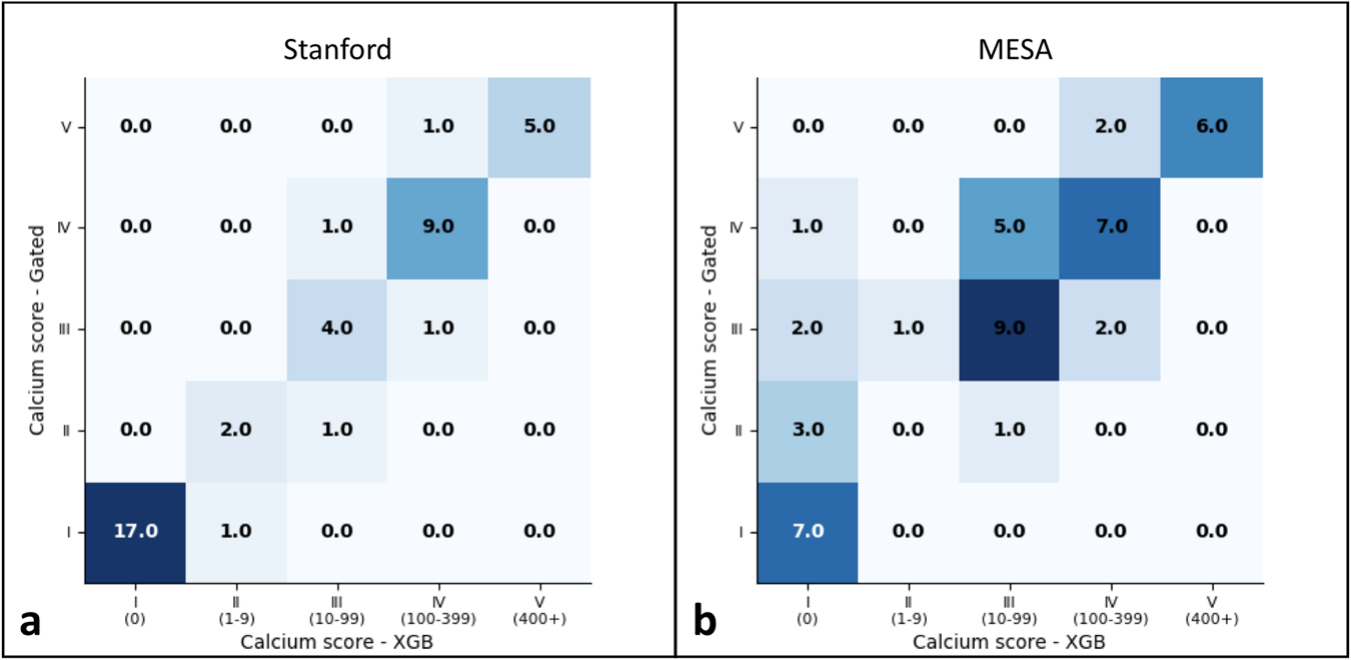
Comparison of automated and manual CAC scoring on non-gated chest CT exams using internal and MESA datasets. Confusion matrices for Stanford (**a**) and MESA (**b**) test sets comparing automated scoring non-gated chest CT exams to ground truth scores. Ground truth scores are on the *y*-axis and model prediction are on the *x*-axis of each matrix.

We trained a non-gated deep learning model on an internal dataset comprised of non-gated routine chest CT studies acquired at Stanford Hospital and those from the Multi-Ethnic Study of Atherosclerosis (MESA) study^33^. We evaluated the model on two test sets: 42 chest CT exams in 42 patients from Stanford and 46 chest CT exams in 46 patients from MESA (Table 2) who also underwent a paired gated coronary CT which served as a reference standard for the CAC score. When comparing bucketed CAC scores, Cohen’s Kappa statistic showed almost perfect agreement between automated and manually derived CAC scores for the Stanford test set (Kappa = 0.84, *P* < 0.0001) and moderate agreement for the MESA test set (Kappa = 0.52, *P* < 0.0001) (Table 3 and Fig. 5). Recent cholesterol guidelines recommend initiating statin therapy when CAC score is ≥100 to reduce future risk of CVD events^34^. For binary classification of patients with CAC score greater than or equal to 100, the non-gated model had a sensitivity and positive predictive values (PPV [95% CI]) of 94 [86, 100]%. For the MESA test set, the model had a sensitivity of 71 [58, 85]%, and a PPV of 88 [79, 98]% for detecting CAC of ≥100. For detecting the presence of any CAC, sensitivity and PPV were high on the Stanford dataset (100% and 96 [90, 100]%). Sensitivity for any CAC was lower on MESA dataset (85 [74, 95]%) but a higher PPV (100%) was seen. Model performance at other CAC score cutoffs are shown in Table 3.

**Table 3:** Diagnostic performance of non-gated model.

Cutoff CAC score	Metric^a^	Internal validation cohorts		External validation cohorts			
		Stanford	MESA	Site 1	Site 2	Site 3	Site 4
	Cohen’s Kappa	0.836	0.517	0.802	0.684	0.644	0.583
1	Sensitivity (%)	100 (100, 100)	84.6 (74.2, 95.0)	94.4 (84.9, 100)	92.5 (86.5, 98.4)	93.0 (87.0, 99.0)	81.9 (75.4, 88.4)
	Specificity (%)	94.4 (87.5, 100)	100 (100, 100)	100 (100, 100)	90.9 (84.4, 97.4)	78.6 (68.9, 88.3)	90.4 (85.4, 95.4)
	PPV (%)	96.0 (90.1, 100)	100 (100, 100)	100 (100, 100)	96.1 (91.7, 100)	87.0 (79.0, 94.9)	93.2 (88.9, 97.4)
	NPV (%)	100 (100, 100)	53.8 (39.4, 68.3)	80.0 (63.3, 96.7)	83.3 (74.9, 91.8)	88.0 (80.3, 95.7)	75.8 (68.6, 83.0)
	F1	0.980	0.917	0.971	0.942	0.899	0.872
10	Sensitivity (%)	100 (100, 100)	88.6 (79.4, 97.8)	100 (100, 100)	95.9 (91.4, 100)	92.3 (86.0, 98.6)	88.9 (83.6, 94.2)
	Specificity (%)	95.2 (88.8, 100)	90.9 (82.6, 99.2)	100 (100, 100)	88.5 (81.2, 95.7)	84.4 (75.8, 92.9)	90.5 (85.5, 95.4)
	PPV (%)	95.5 (89.2, 100)	96.9 (91.8, 100)	100 (100, 100)	94.0 (88.6, 99.4)	87.8 (80.1, 95.5)	91.4 (86.7, 96.2)
	NPV (%)	100 (100, 100)	71.4 (58.4, 84.5)	100 (100, 100)	92.0 (85.9, 98.1)	90.0 (82.9, 97.1)	87.7 (82.2, 93.2)
	F1	0.977	0.925	1.000	0.949	0.900	0.901
100	Sensitivity (%)	93.8 (86.4, 100)	71.4 (58.4, 84.5)	92.9 (82.1, 100)	90.6 (84.0, 97.2)	88.5 (80.9, 96.0)	74.0 (66.6, 81.4)
	Specificity (%)	96.2 (90.3, 100)	92.0 (84.2, 99.8)	100 (100, 100)	93.0 (87.3, 98.8)	93.3 (97.4, 99.2)	94.1 (90.1, 98.1)
	PPV (%)	93.8 (86.4, 100)	88.2 (78.9, 97.5)	100 (100, 100)	90.6 (84.0, 97.2)	88.5 (80.9, 96.0)	88.1 (82.6, 93.6)
	NPV (%)	96.2 (90.3, 100)	79.3 (67.6, 91.0)	88.9 (75.8, 100)	93.0 (87.3, 98.8)	93.3 (97.4, 99.2)	86.0 (80.2, 91.9)
	F1	0.938	0.789	0.963	0.906	0.885	0.804
400	Sensitivity (%)	83.3 (72.1, 94.6)	75.0 (62.5, 87.5)	100 (100, 100)	93.8 (88.3, 99.2)	91.7 (85.1, 98.2)	83.3 (77.0, 89.6)
	Specificity (%)	100 (100, 100)	100 (100, 100)	91.7 (80.2, 100)	93.2 (87.5, 98.9)	98.3 (95.3, 100)	97.4 (94.8, 100)
	PPV (%)	100 (100, 100)	100 (100, 100)	90.9 (78.9, 100)	78.9 (69.7, 88.2)	91.7 (85.1, 98.2)	83.3 (77.0, 89.6)
	NPV (%)	97.3 (92.4, 100)	95.0 (88.7, 100)	100 (100, 100)	98.2 (95.2, 100)	98.3 (95.3, 100)	97.4 (94.8, 100)
	F1	0.909	0.857	0.952	0.857	0.917	0.802

*PPV* positive predictive value, *NPV* negative predictive value.

^a^All values significant (*P* < 0.0001).

### Non-gated chest CT model: external validation

**Fig. 6:**
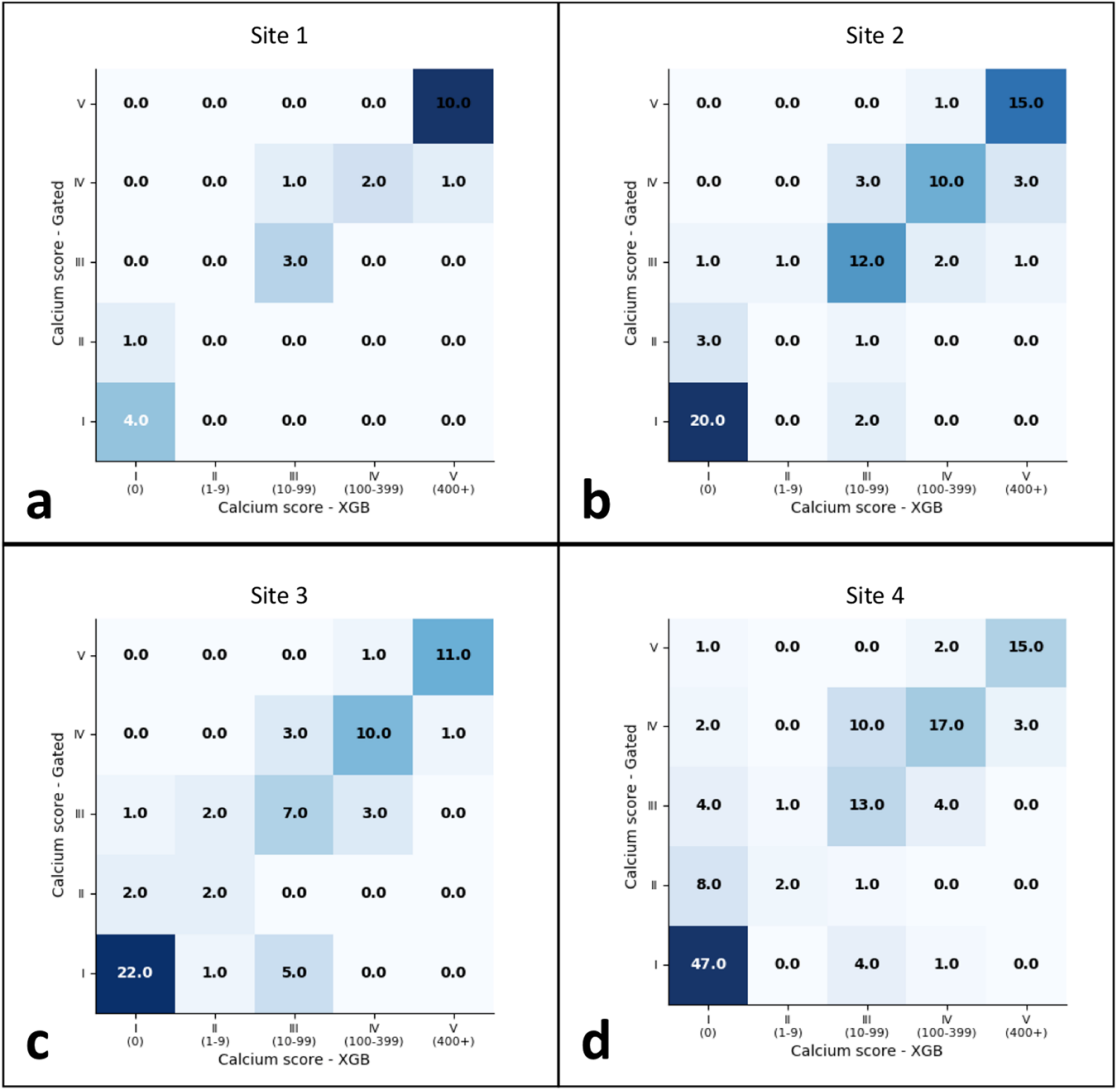
Comparison of automated and manual CAC scoring on non-gated chest CT exams using four external site datasets. Confusion matrices for the four (**a**–**d**) external validation datasets comparing automated scoring non-gated chest CT exams to ground truth scores. Ground truth scores are on the *y*-axis and model prediction are on the *x*-axis of each matrix.

**Fig. 7:**
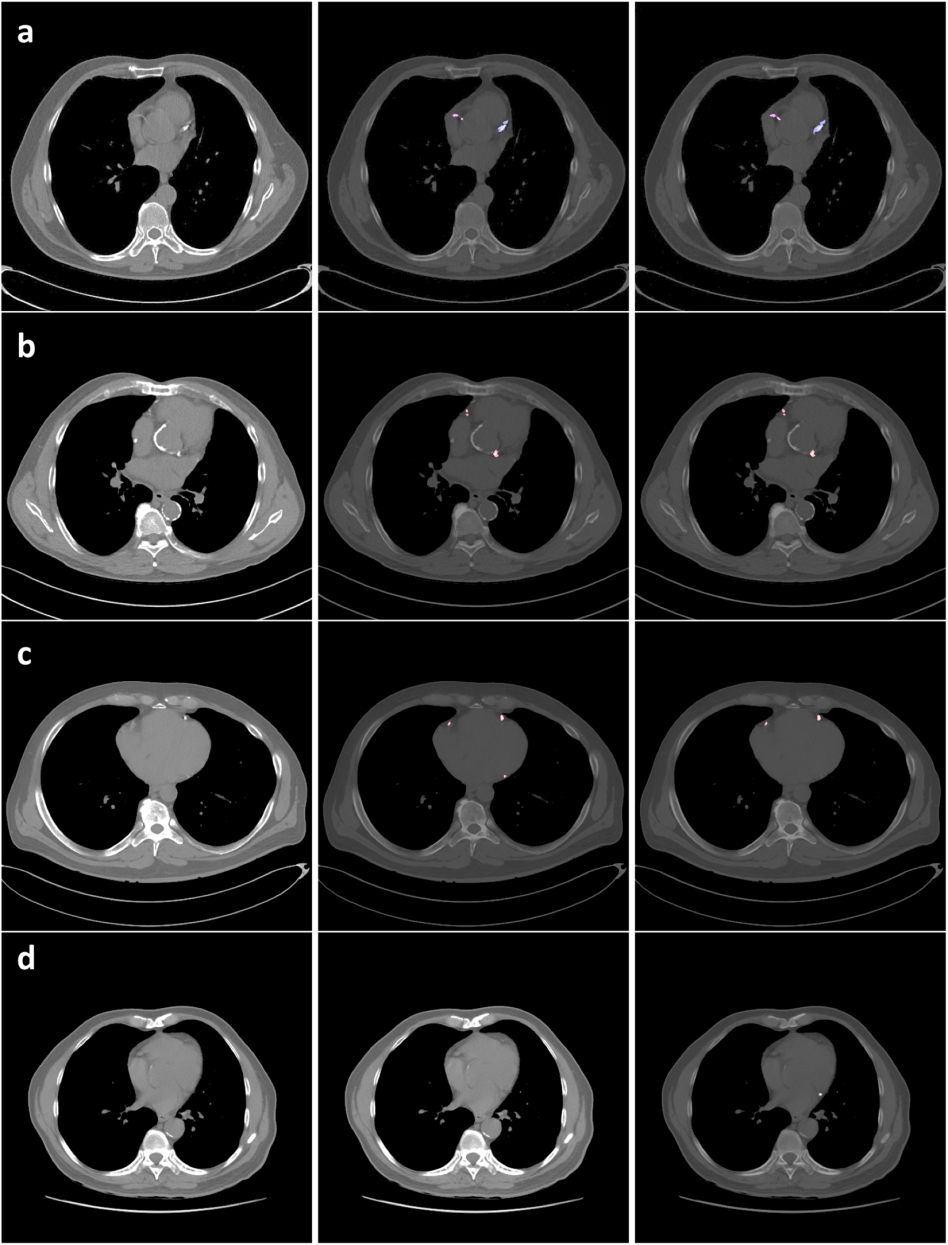
CAC scoring case examples. Coronary artery segmentation (reference image on left, manual in middle; automated on right) in four different patients (**a**–**d**). Row **a** shows qualitatively similar performance between automated and manual methods of CAC scoring on non-gated chest CTs. Row **b** shows model prediction in a patient with significant aortic root calcification. Note that the model does not misclassify this as CAC. Row **c** is an example of false negative prediction by the model in the left circumflex. Row **d** is an example of a false positive in the left circumflex.

Our non-gated model was externally validated on datasets at four geographically disparate sites. Model performance at all sites is summarized in Table 3 and shown in Fig. 6. The F1 score of the model was high at all four external sites (≥0.80). When comparing bucketed CAC scores, Cohen’s Kappa statistic showed substantial agreement at site 1 (Kappa = 0.80, *P* < 0.0001), moderate agreement at sites 2 and 3 (Kappa = 0.68 and 0.64, respectively; *P* < 0.0001), and fair agreement at site 4 (Kappa = 0.58, *P* < 0.0001). Diagnostic performance for detecting any CAC (≥1) was high at all sites (sensitivity range: 82–94% and PPV range 87–100%). Sensitivity and PPV for detecting CAC ≥ 100 was highest at sites 1 and 2 (93 [82, 100]%, 100% and 91 [84, 97]%, 91 [84, 97]%, respectively) (Table 3 and Fig. 7 and Supplementary Fig. 3).

### Diagnostic performance based on ground truth methodology chosen

**Fig. 8:**
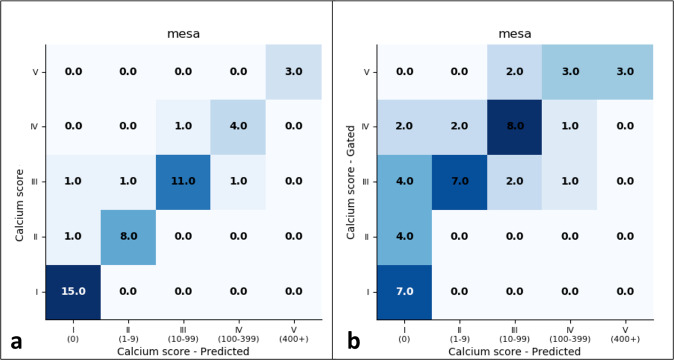
Confusion matrices for non-gated model performance on the MESA test set based on ground truth method chosen. The left matrix (**a**) compares model prediction to scores derived from manual segmentations on non-gated chest CT exams while the right matrix (**b**) compares model predictions to scores derived from gated coronary CT exams, the clinical reference standard for CAC scoring. Note the performance differences (higher on the left) based on ground truth chosen.

Compared to other studies that reported using deep learning to automate CAC scoring on non-gated chest CT exams^26–31^, we chose to use calcium scores derived from paired gated coronary studies as the ground truth for non-gated routine chest CTs. The rationale for this was to ensure accurate quantitation that reflects true calcium burden as defined by the current clinical standard, which is gated coronary CT exams. To highlight the disparity and role of ground truth in diagnostic performance, we report results if ground truth convention used by others were to be followed. That is, we compare our model performance to calcium scores derived from those obtained through manual segmentation by a board-certified diagnostic radiologist on the non-gated routine chest CT exams rather than CAC scores obtained from the contemporaneous gated coronary CTs. For the MESA dataset, individuals underwent the gated coronary CT and non-gated chest CT on the same day and, therefore, serves as an ideal cohort for this experimentation. Figure 8 and Table 4 show the differences in performance in baseline models (without XGBoost) based on the ground truth method used. As an example, baseline model performance for binary classification of CAC ≥ 100 would have an F1 of 0.88 if gated studies were not used as the ground truth (compared to 0.48).

**Table 4:** Diagnostic performance of non-gated model based on chosen ground truth method.

Cutoff score	Non-gated routine chest CT					Gated coronary CT				
	Sensitivity (%)	Specificity (%)	PPV (%)	NPV (%)	F1	Sensitivity (%)	Specificity (%)	PPV (%)	NPV (%)	F1
1	93.5 (86.4, 100)	100 (100, 100)	100 (100, 100)	88.2 (78.9, 97.5)	0.967	74.4 (61.7, 87.0)	100 (100, 100)	100 (100, 100)	41.2 (27.0, 55.4)	0.853
10	90.9 (82.6, 99.2)	100 (100, 100)	100 (100, 100)	92.3 (84.6, 100)	0.952	57.1 (42.8, 71.4)	100 (100, 100)	100 (100, 100)	42.3 (28.0, 56.6)	0.727
100	87.5 (77.9, 97.1)	97.4 (92.7, 100)	87.5 (77.9, 97.1)	97.4 (92.7, 100)	0.875	33.3 (19.7, 47.0)	96.0 (90.3, 100)	87.5 (77.9, 97.1)	63.2 (49.2, 77.1)	0.458
400	100 (100, 100)	100 (100, 100)	100 (100, 100)	100 (100, 100)	1.000	37.5 (23.5, 51.5)	100 (100, 100)	100 (100, 100)	88.4 (79.1, 97.6)	0.545

*PPV* positive predictive value, *NPV* negative predictive value.

## Discussion

In this study, we developed fully automatic, end-to-end deep learning models for automated CAC scoring using gated coronary CT and non-gated routine unenhanced chest CT exams. The novelty and impact of our work is that our models are completely end-to-end, were trained using a stronger reference standard, and the non-gated model was evaluated on multiple external datasets. We also release labeled datasets of gated and non-gated scans with annotations to potentially help fuel further efforts in this domain by other investigators. Our gated model achieved almost perfect agreement with manual CAC scoring and took less time than conventional methods. Our non-gated model achieved good diagnostic performance in identifying patients with any CAC and CAC scores ≥100 across all sites (PPV ranges from 87 to 100%).

CAC is an imaging biomarker of coronary atherosclerotic disease and an independent indicator of future cardiovascular events^35^. A US Preventive Services Task Force (USPSTF) statement provided an evidence report highlighting that adding CAC to traditional risk models results in highest improvement in disease discrimination and risk reclassification compared to other nontraditional factors (e.g., ankle-brachial index and high-sensitivity C-reactive protein)^36,37^. Most notably, a recent large retrospective study determined that the presence and severity of CAC identified patients that would most benefit from statin therapy^38^. Our work relates to improving the efficiency and reducing potential barriers to obtaining CAC scoring. One such barrier may be the physical task of performing the CAC quantitation. For small radiology practices, a single- or small group of radiologists may use an independent workstation to generate CAC score reports^39^. However, this may not be sustainable as those practices grow, nor is it for larger centers with increasing volume and breath of cases. Thus, many centers employ 3D labs where post-processing for cross-sectional imaging, including CAC scoring, is performed^39,40^. Costs associated with these 3D labs often include those associated with space, post-processing hardware and software, and salaries for dedicated specialized technologist^39,40^. Our gated model performs CAC scoring at a nearly perfect level to the ground truth with vessel-specific calcium burden in a fraction of the time required by manual process. Such automation could help decrease costs for hospital systems and help streamline workflow for busy 3D labs that could focus on other complex post-processing tasks.

To date, a few deep learning models for automated CAC scoring on gated coronary CT have been reported. A notable difference and advancement of our deep learning model is that it is fully-automated and end-to-end with vessel-specific calcium scoring, not requiring co-registered CT atlas^23,41^ or a coronary CT angiography to define coronary artery anatomy^25^. Although we did not use the aforementioned techniques used by others to focus the models on relevant coronary anatomy, our model is able to discern between coronary and non-coronary calcification (e.g., valvular calcification) that might otherwise present as a false positive.

Cardiovascular disease (CVD) is the leading cause of death in the United States (US) resulting in annual direct and indirect costs of $320 billion^42,43^. It is projected that by 2030, 44% of the US population will have some form of CVD resulting in an increase to $918 billion^43^. Treatment of risk factors, including the use of anti-hypertensive and lipid-lowering treatment, can significantly impact future incidence of CVD. Studies have shown that presence of CAC appears to motivate an improved diet, increased exercise, and the initiation of and adherence to preventive therapies^44,45^. The latest ACC/AHA cholesterol guidelines recommend testing for CAC when patients are at low to intermediate risk for a heart attack, and when there is uncertainty about whether or not to prescribe a statin medication for cholesterol lowering. When the CAC score is ≥100 Agatston units, these guidelines recommend using statin therapy to reduce risk^34^. However, CAC testing and preventive therapies remain vastly underutilized particularly as most insurance plans do not usually cover CAC testing. As a result, millions of asymptomatic people remain unaware of their high risk for a heart attack and remain undetected and undertreated. Meanwhile, up to 19 million non-gated chest CT scans were performed in 2014 alone for non-cardiac indications^18,46^. Though these exams may demonstrate coronary calcification, up to 80% of radiologist reports do not mention it^47,48^. Even if radiologists report incidental coronary calcification, accurate quantitation is difficult as no widely accepted standard currently exists unlike scoring using gated coronary CT. Thus, calcification burden would be reported subjectively (e.g., mild, moderate, or severe), if at all. This underreporting represents a missed opportunity as the ability to accurately, systematically, and efficiently determine the presence and quantify the severity of CAC from existing ungated chest CTs would allow opportunistic screening of patients for cardiovascular risk without incremental radiation or cost. We developed a deep learning model that can automatically quantify coronary calcium burden on non-gated chest CTs. Using a model such as ours could potentially allow millions of patients at risk for cardiovascular disease to be identified and presented with the opportunity to start preventive medication and lifestyle change to reduce the risk of myocardial infarction. This deep learning model could also provide added value to routine radiologist interpretations via automated quantification and reporting on routine chest CTs. In addition to the large number of patients who receive a chest CT for other indications, this model could also be applied to populations of chest CTs retrospectively to identify high-risk individuals and potentially intervene with optimized medical management, leading to significant advantages for population health prevention management efforts.

Our deep learning model for automated CAC scoring on non-gated chest CTs has some key notable differences to the few other models reported in the literature to date^26,28,41,49^. Some models use a two-stage process for CAC scoring^28^, such as an atlas for registration^41^ or bounding box to define anatomy^49^. Our model uses a single convolutional neural network (CNN) for an end-to-end approach. Most significantly, all deep learning models^26,28,29,31,32^ on CAC scoring using non-gated unenhanced chest CTs reported to date have used manual scoring on non-gated chest CTs solely as the reference standard, which may be inadequate. Most notably, a recent study by Zeleznik et al.^32^ demonstrated substantial agreement between automated and manual stratification of CAC scores into one of four risk buckets in a multi-center trial cohort comprised of over 20k asymptomatic individuals. However, the comparison here was also made to manual quantitation performed on non-gated studies as opposed to the current standard of care to quantify CAC, a gated coronary CT exam. Though several studies^50–55^ have reported good agreement between CAC scores derived from non-gated chest CTs and gated coronary CTs, there were still significant differences in median absolute scores. Moreover, practice convention has not changed, and the current standard clinical practice is to still use gated coronary CTs for accurate CAC scoring^56^. A number of factors may contribute to this lack of paradigm shift^57^. The most obvious is the superior spatial resolution of coronary CT and lack of motion artifact from ECG-gating thereby minimizing over- and underestimation of calcium that might otherwise occur with routine non-gated chest CTs^16,50,58^. Because non-gated chest CT are performed for non-cardiac indications, acquisition parameters differ than those for gated coronary CTs which can affect the accuracy of calcium quantitation^59^. Coronary CTs for calcium scoring are often reconstructed with a smaller field-of-view, higher definition kernel, and thinner slice thickness (e.g., 2–3 mm) compared to routine non-gated chest CTs which are typically reconstructed with a soft tissue kernel and thicker collimation (e.g., 5 mm)^16^. While lung cancer screening non-gated chest CT and low dose gated coronary CT may have similar radiation doses (i.e., 30–35 mAs)^60,61^, standard non-gated chest CT doses for non-lung cancer screening can approach tube currents of 200 mAs^61^. These tube current differences can affect image noise and quantitation^62^. Thus, we used Agatston scores from gated coronary CT as a more robust reference standard for our non-gated model training in an attempt to more accurately quantify calcium burden on non-gated chest CTs. We further highlight the significance of an adequate ground truth by reporting diagnostic performances between our method that used gated CTs and those used by others that use human annotations on non-gated routine chest CTs. This analysis revealed that results of our model performance would have been inflated if an inadequate clinical reference standard, in this case, a score derived from segmentations using a non-gated chest CT, were used. The need for accuracy is particularly significant in light of the latest cholesterol guidelines recommending statin therapy initiation in patients with a CAC ≥ 100^34^.

This study has important limitations. For both models, limitations associated with a retrospective study design are present. For the gated model, the input requires a reconstructed smaller-field-of-view (FOC) around the heart. Thus, centers that do not routinely reconstruct smaller FOV would have to make this additional exam processing step prior to using such a model for inference. For the non-gated model, we used gated coronary CTs as the reference standard for Agatston scores on corresponding chest CTs; however, the paired scans were performed at different times except for the MESA cohort. Therefore, the interval difference between the gated and non-gated scan could affect the accuracy of scores used for the non-gated chest CTs. We used a maximum time interval of 1 year for all test sets. Studies have shown that a majority of patients with zero CAC scores on CT show no annual increase^63^. However, patients with existing CAC may show progression, and thus, it is possible some of our patients may have progressed. An annual change of 8.3 Agatston unit change has been reported for patients with baseline scores of 1–100^63^; thus, the impact on the ungated model’s performance on classifying patients with a score of ≥100 may not be significant. Finally, because our training data would not likely have a sufficient number of training example of patients with anomalous coronary arteries, the models would not be expected to perform reliably in such cases.

In conclusion, we developed deep learning models capable of performing CAC scoring using both gated coronary CT and non-gated chest CT. These models could potentially reduce barriers for screening larger populations and thereby allow initiation of preventive therapy such as statin use. Further studies are necessary for correlating automated scores to patient outcomes.

## Methods

All site protocols were Health Insurance Portability and Accountability Act-compliant and approved by respective Institutional Review Board of the participating institutions, and a waiver of informed consent obtained. All sites participated as a consortium and external validation was performed through a federated manner in which no data was shared between sites.

### Retrospective internal coronary CT dataset

We retrospectively searched the electronic medical record database at our single tertiary care academic center (Stanford Hospital and Clinics) for consecutive patients who underwent prospectively gated unenhanced coronary CT for CAC scoring between June 2016 and July 2018. 866 CT exams in 854 unique patients were collected (Table 1). 5 mm small field-of-view (FOV) axial slices were used as input for deep learning model development. The smaller FOV is routinely reconstructed at our institution as part of routine clinical practice. These studies were split into training (708 studies in 697 patients), validation (79 studies in 78 patients), and test sets (79 studies in 79 patients). To generate the validation and test sets, stratified random sampling was used to ensure that there was class balance. There was no patient overlap between training, validation, and test sets. Ground truth scores were those that were performed manually and prospectively at the time of the clinical exam and were extracted from the radiology reports. Automated CAC scores generated by the deep learning model were then compared to these manual derived scores. Vendor and scanner model distribution is reported in Table 1.

### Prospective internal coronary CT cohort

For internal prospective validation of our gated deep learning CAC scoring model, we collected 55 unenhanced gated coronary CT exams performed on 55 unique patients over a 5-day trial period in September 2018 (Table 1). Exams were retrieved from our Picture Archiving and Communication System (PACS) after image acquisition at the end of each day. Automated CAC scores generated by the deep learning model were compared to manually derived scores from three independent 3D lab technologists. One technologist labeled the exam prospectively at the time of the clinical exam, working in parallel with a radiologist who is responsible for interpreting the entire exam as part of our standard clinical workflow^40^. Two technologists, with 7 and 10 years of experience, retrospectively annotated the scans for calcium scoring. Vendor and scanner model distribution is reported in Table 1.

### Internal chest CT cohort

**Fig. 9:**
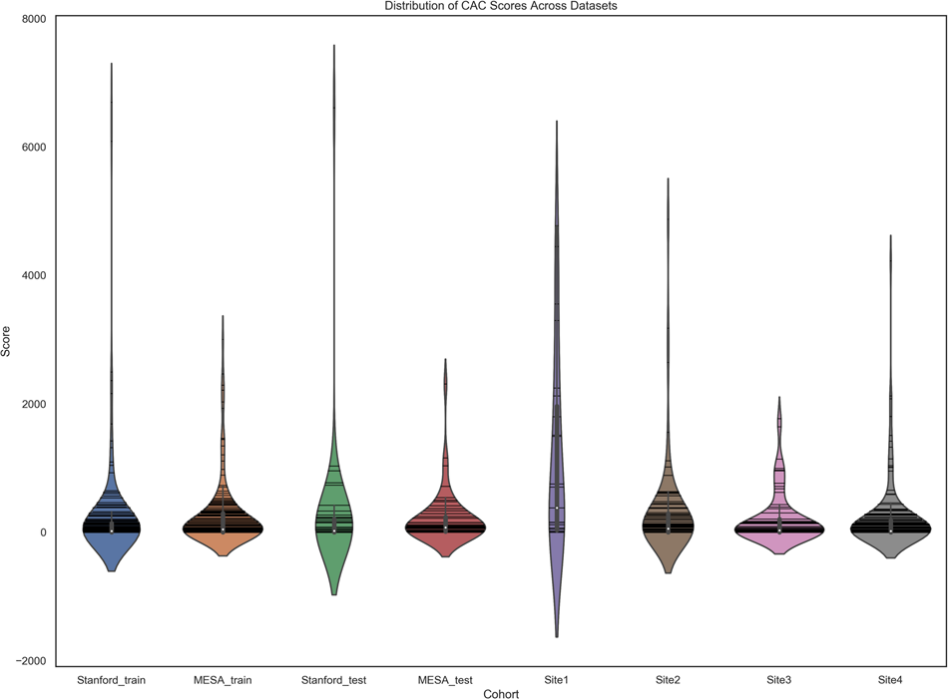
CAC score distribution across internal and external sites used for the non-gated model. Violin plots show median (white point), each data point (horizontal lines), and interquartile range (vertical gray bar). Note that though the plots extension below a CAC score of 0 reflects properties of the kernel density estimator associated with violin plots. CAC score of less than 0 would not be realistic.

The non-gated model was trained on a mixed cohort dataset of data from Stanford and that from the MESA study. First, we retrospectively searched our PACS for patients who underwent a non-gated unenhanced chest CT and a gated coronary CT between 2013 and 2018. The gated coronary study served as the reference standard for CAC scores on ungated chest CTs. To increase the size of the internal training datasets, patients were eligible if a gated coronary CT was performed within no more than a 2-year time interval of the index chest CT exam, though the test set (*n* = 42) comprised of only paired studies that were no more than one year apart (median time interval of 3.3 months). Rationale for restricting the test set to 1 year was based upon studies showing 8.3 Agatston unit change for patients with baseline scores of 1–100^63^ and an overall low percentile (~5%) annualized change or median progression (29 Agatston units/year) in calcium score^63,64^. MESA non-gated chest CTs and MESA gated coronary CTs were performed on same day. A random 70/10/20 split to create training, validation, and test sets was performed. Table 2 shows the data distribution (Fig. 9), cohort statistics, and time interval between the paired exams. A total of 447 exams were included between Stanford and MESA for training (Table 2). 5 mm axial slices from unenhanced chest CTs were used as input with Agatston scores from corresponding gated coronary CT exams as output for model development. Automated CAC scores generated by the deep learning model on non-gated routine CT chest exams were compared to manually derived scores from gated coronary exams.

### External site descriptions

External datasets from 4 sites were obtained in which retrospectively performed paired exams no more than 1 year apart were included. The median time interval between exams and other data statistics are shown in Table 2. Distribution of CAC scores across the sites are illustrated in Fig. 9. Site 1 represented an inpatient and outpatient health system affiliated with a University. Site 2 represented the largest diagnostic radiology company in Latin America (5th largest in the world). Sites 3 and 4 represented major tertiary academic centers.

### Reference standard, annotation, and image preprocessing

For both the coronary CT and chest CT cohorts, a board-certified radiologist manually segmented calcium within the four coronary arteries on slice-by-slice level using an open-source Digital Imaging and Communications in Medicine (DICOM) viewer (Horos version 3.1.1, 2019 The Horos Project). These segmentation masks were used for model training. Ground truth calcium scores corresponding to these segmentations were taken from were derived from the exam report that was generated prospectively at the time of image acquisition. Per routine clinical workflow, scores were calculated using established methods using the 130 Hounsfield Unit (HU) threshold^65^. For chest CT exams, while reports may provide a qualitative score (e.g., mild, moderate, or severe coronary calcification), quantitative scores are not provided and are not standard of care. Because of this and because use of the conventional 130 HU threshold may be insensitive to detect calcium within voxels due to inherent differences in acquisition parameters for non-gated chest CT compared to gated coronary CTs^59^, Agatston scores from the corresponding coronary CT exam was used as ground truth for the segmentations. Exams for input into the model were extracted from our PACS in DICOM format and scaled to 512 × 512 pixels.

### Algorithm

Our approach was to develop a fully automatic algorithm that takes a CT exam series as input, and outputs an Agatston score for each of four coronary arteries: LCA, LAD, LCX, and RCA. This approach requires neither manual segmentation of anatomy nor a cardiac atlas for localizing the coronary arteries, and thus contrast-enhanced coronary CT angiography (CTA) is not required.

The first stage of our algorithm is a convolutional neural network (CNN) which takes a CT volume as input and processes it slice-by-slice. The CNN performs two operations end-to-end: First, it segments calcium lesions that contribute to the Agatston score. Second, the CNN categorizes each calcium lesion as belonging to the LCA, LAD, LCX, or RCA. We choose to parametrize these two functions with the same neural network because both functions require significant knowledge of the underlying coronary anatomy. For example, the model must be able to localize the aorta to avoid segmenting irrelevant calcium lesions, since calcium lesions inside the aorta do not contribute to the Agatston score. Similarly, mitral and aortic valvular calcifications must be localized so as not to inadvertently incorporate them into the final CAC score.

Concretely, for each pixel of the input, the CNN outputs a vector of probabilities (*p_calc,p_lca,p_lad,p_lcx,p_rca*), where *p_i* ∈ (0,1) for all i and *p_lca* + *p_lad* + *p_lcx* + *p_rca* = 1. The second stage of our algorithm is tasked with converting from these pixel-by-pixel probabilities to the Agatston score contributed by a slice. We begin by classifying all pixels with *p_calc* > 0.5 as belonging to a calcified lesion. We perform connected components analysis with 8-connectivity to combine pixels into calcium lesions. Further, we assign to each pixel the artery label given by argmax (*p_lca,p_lad,p_lcx,p_rca*) and we label the entire lesion with the most frequently occurring label assigned to its constituent pixels. Finally, we calculate the area of each calcium lesion, restricting to pixels with an attenuation of >130 Hounsfield Units, and scale the area by the maximum attenuation. The slice-wise Agatston scores are summed to arrive at the Agatston score for the CT exams

For all experiments on ungated exams, an additional post-processing stage was applied. The segmentation outputs were summarized using vector with four components: the sum of predicted calcified area, the maximum intensity in HU within the predicted calcified region, sum of area weighted by maximum intensity within each lesion, and the number of lesions predicted. These summary vectors were then used as input to a gradient boosted decision tree classifier. In particular, summary vectors were computed for each axial slice in the exam, and the summary vectors for the slices with the 5 highest predicted areas were concatenated. The resulting 20-component vector was used as input to a gradient boosted decision tree classifier which was trained and cross-validated using the same dataset splits as the CNN, using CAC scores from paired gated coronary CT as a reference standard. The CAC scores from paired gated coronary CT were also used as a reference standard for evaluation of studies in the test set. The gradient boosted decision tree classifier was trained to classify entire exams into CVD risk buckets I–V.

### CNN architecture

**Fig. 10:**
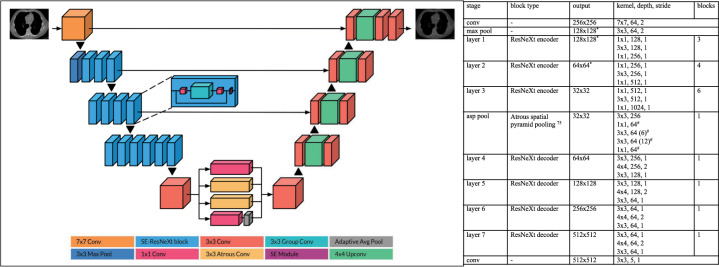
Convolutional neural network architecture^77^. *Output used as a skip connection. ^#^Parallel blocks.

Both the gated coronary model and non-gated chest CT model were trained with the same CNN architecture on different datasets. For both models, we used an encoder-decoder architecture, where the encoder is a 50-layer SE-ResNeXt 2D CNN^66^ pretrained on ImageNet^67^ (Fig. 10). We employed skip connections at three levels from the encoder to the decoder, in a fashion similar to U-Net^68^. The decoder is a stack of 4 convolutional blocks, where each block performs 2× up-sampling with a 4 × 4 transposed convolution, sandwiched between two 3 × 3 convolutions.

We trained our network on 4 NVIDIA GeForce GTX 1070 GPUs, using input slices of raw Hounsfield Units clipped to the range [−800,1200] and zero-centered. During training, we randomly sampled mini-batches of 64 slices containing calcified lesions. We used the Adam optimizer with default β parameters (β1 = 0.9, β2 = 0.999) and a learning rate of 1 × 10^(−3) for the randomly initialized weights and 1 × 10^(−4) for the pretrained weights. Training began by only including slices with calcium lesions, and we expanded the training set after every epoch to include misclassified slices with no calcified lesions. For the learning rate schedule, we adopted a linear warmup for 5k iterations, followed by cosine annealing for 300k iterations^69,70^. Additionally, we delayed the learning rate schedule by 10k iterations for the pretrained weights. We applied L2 regularization of 1 × 10^(−4) to all learnable parameters. For the loss function, we used cross-entropy loss for the region-wise classification head, and Dice loss^71,72^ for the binary segmentation (calcium vs. no-calcium) head. At test time, we sequentially sample slices from each series, and we sum the region-wise predictions for each slice to obtain the series-level Agatston score.

### Statistical analysis

Statistical analysis was performed using the Statistical Analysis System (SAS) software, version 9.4 (SAS institute, Cary, NC). To compare individual vessel scores between automated model and human readings, Bland–Altman Plot was used to evaluate the agreement. Through Bland–Altman Plot, the average bias and its 95% confidence intervals allows quantification and the range of differences between automated and manually derived scores^73^. Kolmogorov–Smirnov (K–S) test was also used to analyze the similarity of distributions from the deep learning model predictions and human scores. For bucketed scores (I–V, for Agatston scores of 0, 1–10, 11–100, 101–400, >400, respectively^74,75^), Cohen’s Kappa statistics was used to evaluate the level of agreement between the automated and manually derived scores. The following guidelines for the interpretation of Kappa coefficients were used: (<0.00): poor; (0.00–0.20): slight; (0.21–0.40): fair; (0.41–0.60): moderate; (0.61–0.80): substantial; (0.81–1.00): almost perfect agreement^76^. Kappa statistics were also visualized with the agreement plots from which we can show the distribution of each categories (the size of the bucket) and the agreement status (exact agreement: dark blue, partial agreement (differ from 1 between two methods): light blue, and complete disagreement: blank). To address a large amount of zero values and abnormal distribution in CAC scores, for the prospective trial, a random effect zero-inflated Poisson regression model was used to compare model CAC scores to the three manual readings. Diagnostic performance metrics (sensitivity, specificity, PPV, NPV, and F1) at various CAC score cutoffs were calculated for the non-gated model.

### Reporting summary

Further information on research design is available in the Nature Research Reporting Summary linked to this article.

## Supplementary information

Supplementary Information

Reporting Summary

## Data Availability

Gated coronary CTs and the accompany segmentations calcium scores along with paired gated and non-gated routine chest CTs with calcium scores is available at https://stanfordaimi.azurewebsites.net/datasets/e8ca74dc-8dd4-4340-815a-60b41f6cb2aa.

## Code availability

Restrictions apply regarding the use of source code used in this study. However, it can be made available from the corresponding author (B.P.) upon reasonable request(s).
