# Supplementary material for: Automated coronary calcium scoring using deep learning with multicenter external validation
Source: NPJ Digit Med. 2021 Jun 1;4:88. doi: 10.1038/s41746-021-00460-1 (PMC8169744; doi:10.1038/s41746-021-00460-1)
Supplement: Supplementary file 1 — Supplementary Information [file 41746_2021_460_MOESM1_ESM.pdf]

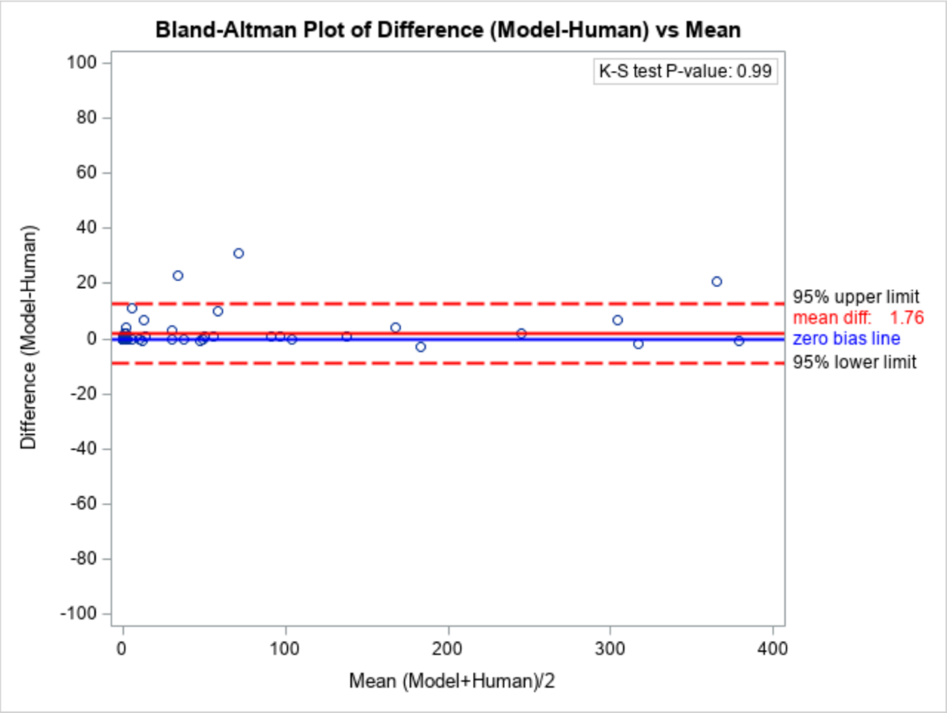

Supplementary Figure 1. Bland-Altman plot comparing automated to manual scoring of CAC on gated coronary CT exams of CAC score ≤ 400.

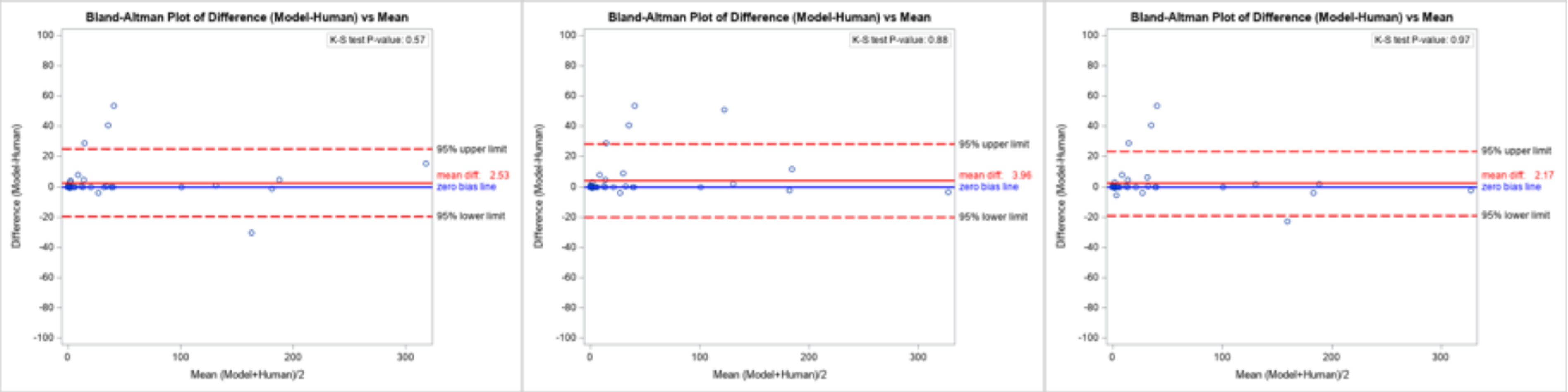

Supplementary Figure 2. Bland-Altman plots comparing automated scoring to manual scoring by three different (left, middle, right) technologists on gated coronary CT exams of CAC scores ≤ 400.

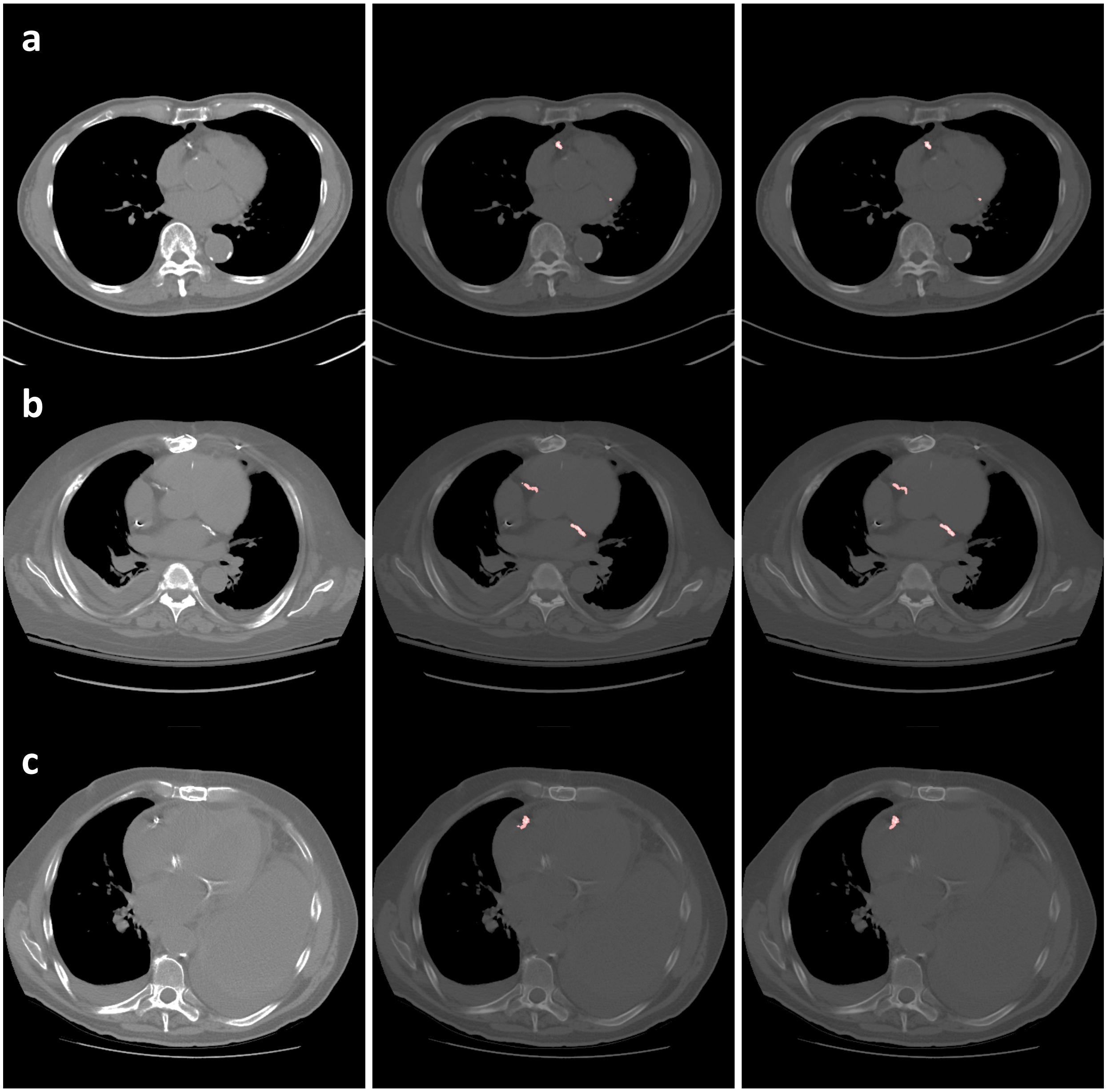

Supplementary Figure 3. Coronary artery segmentation (reference image on left, manual in middle; automated on right) in three different patients (rows). Row a shows aortic root calcification (short arrow) and descending aortic calcification (long arrow) that are not inadvertently picked up by the model as coronary calcium. Row b shows the caudal tip of a central venous line (arrow) that is not mistakenly picked up by the model as CAC. Row c is an example of mitral valve calcification (arrow) not inadvertently picked up by the model as CAC.
